# High-Z Contrast Media for Coronary Photon-counting Detector CT Angiography: Improved Quantification of Calcified Stenoses

**DOI:** 10.1097/RLI.0000000000001262

**Published:** 2025-12-01

**Authors:** Tristan T. Demmert, Konstantin Klambauer, Bernhard Schmidt, Victor Mergen, Lukas J. Moser, Philipp N. Maintz, Thomas Allmendinger, Thomas Flohr, Hubertus Pietsch, Matthias Eberhard, Hatem Alkadhi

**Affiliations:** Institute of Diagnostic and Interventional Radiology, University Hospital Zurich, University of Zurich, Zurich, Switzerland (T.T.D., K.K., V.M., L.J.M., P.N.M., T.F., M.E., H.A.); Siemens Healthineers AG, Forchheim, Germany (B.S., T.A., T.F.); Department of Radiology and Nuclear Medicine, Maastricht University Medical Centre+, Maastricht, The Netherlands (T.F.); Bayer AG, Berlin, Germany (H.P.)

**Keywords:** high-Z contrast media, coronary stenosis, calcification, plaques, phantom

## Abstract

**Background:**

Blooming artifacts from calcified plaques can obscure the vessel lumen, leading to overestimation of stenosis severity. Spectral coronary angiography with photon-counting detector CT (PCD-CT) provides virtual monoenergetic images (VMIs) for coronary artery disease assessment. While VMIs at high VMI energy levels reduce calcium blooming, iodine contrast is diminished, limiting diagnostic value. This study evaluated whether contrast media with an atomic number higher than iodine (high-Z) preserve vascular contrast using high VMI energy levels, thereby improving the accuracy of stenosis quantification.

**Methods:**

A phantom with 4 and 6 mm diameter rods to mimic small diameter vessels containing eccentric calcified plaques causing 25%, 50%, and 75% diameter stenoses was scanned with a dual-source PCD-CT system. Five different contrast media, including iodine, tungsten, holmium, hafnium, and bismuth, were tested. VMIs were reconstructed from 40 to 190 keV in 1-keV steps. Vessel attenuation, contrast-to-noise ratio (CNR), and stenoses were measured. Qualitative assessment of image quality was performed.

**Results:**

Iodine attenuation was high at lower VMI energy levels and dropped below 250 HU at >100 keV. Tungsten, holmium, hafnium, and bismuth maintained >250 HU attenuation throughout the entire energy range. Vessel CNR of iodine was high at lower and decreased at higher VMI energy levels, similar to the CNR of holmium and bismuth, though to a lesser extent. In distinction, CNRs of tungsten and hafnium were lower at lower VMI energy levels and increased to a relatively constant level at higher keV. Tungsten CNR increased with energy, approaching ~40 at high keV. Across all contrast media and stenosis degrees, stenoses were overestimated on low VMI energy levels (24% to 32.5% at 40 keV), while the degree of overestimation decreased at higher VMI energy levels (0% to 13.5% at 190 keV). At 190 keV, tungsten, hafnium, and bismuth showed ≤2.5% stenosis overestimation, compared with iodine (10% to 13.5%). Image quality varied between contrast media and energy levels: new very high-Z contrast media achieved higher scores, while iodine peaked at lower keV (55 to 70 keV) and, due to loss of contrast at higher energies, received the lowest overall scores.

**Conclusions:**

As compared with iodine, very high-Z contrast media enable superior lumen definition and more accurate stenosis assessment, also at high VMI energy levels, which minimize calcium blooming.

Coronary CT angiography (CCTA) is the primary noninvasive imaging modality in patients with low to intermediate risk of coronary artery disease.^1^ Still, accurate quantification of coronary stenoses remains challenging in the presence of calcified plaques.^2,3^ Calcium blooming, caused by partial volume effects and beam hardening, can obscure the vessel lumen and lead to overestimation of stenosis severity, potentially affecting patient management and necessitating additional downstream testing.^4,5^

Virtual monoenergetic images represent the routine reconstruction for image analysis of recently introduced photon-counting detector computed tomography (PCD-CT).^6,7^ Using refined image processing,^8^ VMIs provide higher iodine contrast-to-noise ratios (CNR) at lower keV for improved visualization of iodine-enhanced structures. However, blooming artifacts from calcified coronary plaques are largest at these low VMI energy levels, while high energy levels are associated with fewer blooming artifacts.^9^

Unfortunately, high-energy level VMIs result in low to insufficient iodine contrast, compromising the ability to detect and accurately assess luminal narrowing. This trade-off between reduced blooming and diminishing iodine signal remains an issue in routine clinical practice, precluding the use of high VMI energy levels, particularly in CT angiography.^9^ Approaches of addressing this problem by developing personalized contrast injection protocols aimed at optimizing intravascular attenuation at high energy levels VMI^10^ involve higher iodine delivery rates for more severe calcifications, enabling the reconstruction of high energy level VMI with sufficient lumen attenuation. However, this might contradict the *as-low-as-reasonably-achievable* (ALARA) principle in radiology regarding the use of contrast media.

A promising approach to this issue would be the use of novel contrast media with higher atomic numbers (*very high-Z materials*), which exhibit K-edge attenuation characteristics at higher photon energies similar to those of iodine at lower energy levels.^11^ As recently indicated,^12–15^ such experimental contrast media might preserve or even enhance vascular attenuation in high VMI energy levels, potentially enabling both blooming reduction and adequate vessel enhancement without necessitating the administration of larger volumes of contrast media.

To investigate this concept under controlled conditions, we conducted a coronary artery phantom study with calcified, predefined stenoses on PCD-CT, comparing conventional iodinated contrast with the experimental very high-Z agents tungsten, bismuth, hafnium, and holmium. Virtual monoenergetic images at high keV were used to assess vascular attenuation and the accuracy of stenosis quantification.

## METHODS

### Phantom Setup

**FIGURE 1:**
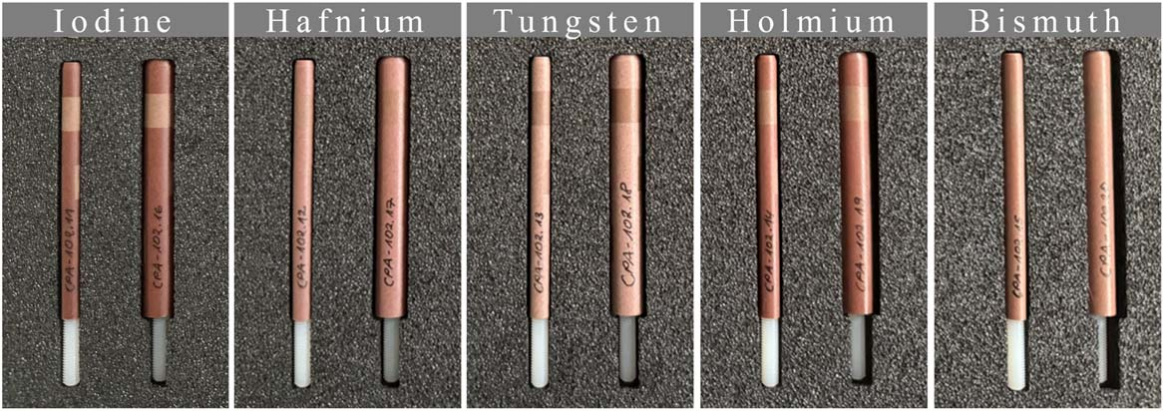
Picture showing the custom-made phantom with the various contrast media and with 2 different vessel sizes.

A custom-made coronary artery phantom [article number: QEM-99100 (B24043)/ serial number: CPA-102.1-.20, QRM GmbH] was designed to mimic small diameter vessels with defined stenoses caused by calcified plaques (Fig. 1). The setup contained 10 vessel stenosis phantoms combining 5 different contrast media in 4 and 6 mm diameter tubes. Each phantom contained 3 different eccentric calcium hydroxyapatite (CaHA) plaques causing 25%, 50%, and 75% diameter stenosis. The plaques consisted of CT water-equivalent enriched with CaHA with an attenuation of 794 HU at 120 kVp. The phantom was placed in a 25×35 cm container filled with oil to simulate the x-ray attenuation characteristics of epicardial fat surrounding the coronary arteries and to approximate the attenuation profile of an adult human chest.

### Contrast Media

In addition to iodine (I), the following high-atomic-number contrast media were included: tungsten (W, atomic number 74), holmium (Ho, atomic number 67), hafnium (Hf, atomic number 72), and bismuth (Bi, atomic number 83). These elements were selected based on their distinct K-edge energies, which influence their energy-dependent attenuation profiles, with K-edges located within the diagnostic x-ray spectrum (∼35 to 100 keV) (Table 1). All contrast media were embedded in a CT water-equivalent base material enriched with the respective elements, mimicking realistic intraluminal contrast conditions.

**TABLE 1:** Contrast Media Characteristics and Stenosis Assessment at 40 and 190 keV

			Attenuation (HU)		Contrast-to-Noise Ratio		75%-Stenosis (in %)		50% Stenosis (in %)		25% Stenosis (in %)	
	Atomic number^11^	K-edge (keV)^11^	40 keV	190 keV	40 keV	190 keV	40 keV	190 keV	40 keV	190 keV	40 keV	190 keV
Bismuth	83	90.5	747	438	35	32	100.0	77.0	81.5	52.5	53.0	26.0
Hafnium	72	65.3	454	522	24	37	99.0	76.0	80.5	51.5	52.5	25.5
Holmium	67	55.6	1166	280	51	22	100.0	79.0	82.0	58.5	54.0	32.5
Tungsten	74	69.5	295	554	19	39	99.0	75.0	81.0	50.0	52.0	25.0
Iodine	53	33.2	1722	74	71	9	100.0	85.0	82.5	63.5	55.5	38.5

To ensure comparable baseline vascular contrast, all mixtures were standardized to achieve ~500 HU at 120 kVp in a water-equivalent matrix. Because the high-Z elements were incorporated as oxides/salts with different mass attenuation coefficients and stoichiometry, the elemental mass fractions required to reach ~500 HU differed: iodine 0.98%, tungsten 1.76%, holmium 1.55%, hafnium 1.65%, and bismuth 2.05% (by mass, element basis). The corresponding measured vessel attenuations at 120 kVp were tightly clustered (I 497 HU; Hf 516 HU; W 520 HU; Ho 516 HU; Bi 520 HU). This attenuation-matching approach controlled the baseline contrast at a clinically relevant level while allowing energy-dependent differences between contrast materials to be assessed on VMI. This attenuation was measured with a single-source energy-integrating detector CT (SOMATOM Definition AS, Siemens Healthineers).

### CT Data Acquisition and Image Reconstruction

All scans were performed using a first-generation dual-source photon-counting detector CT system (NAEOTOM Alpha, Siemens Healthineers; software version VB10), operated with a clinical spectral, prospectively electrocardiography (ECG)-gated cardiac CT protocol with an artificial ECG at 60 bpm. Acquisition parameters were kept constant for all scans: tube voltage 140 kVp, image quality (IQ) level 44, and collimation of 96×0.2 mm.

Scans were reconstructed as vascular spectral post-processing (VSPP) series from ECG-triggered acquisitions, applying a quantitative Qr40 kernel, at a slice thickness of 0.4 mm, an increment of 0.2 mm, with quantum iterative reconstruction (QIR) level 3. The field of view was 200×200 mm^2^, and the matrix was 512 × 512 matrix. Submillimeter images were reconstructed with a slice thickness of 0.4 mm (increment 0.2 mm) to minimize partial volume averaging and to sharpen the lumen-calcification interface for stenosis assessment. Quantum iterative reconstruction at a level of 3 was applied to stabilize image noise.

### Image Analysis

Image evaluation was performed using freeware software (ImageJ, National Institutes of Health). VMIs were reconstructed from VSPP series from 40 to 190 keV in 1-keV increments. The attenuation of the individual contrast media was analyzed across all VMI energy levels using the dual-energy workflow of syngo.via (Siemens Healthineers). Vessel and background attenuation were measured within this application, and a threshold of 250 HU was defined as the minimally required vascular attenuation, as previously recommended.^16^ To determine the contrast-to-noise ratio (CNR) between each contrast medium and the surrounding matrix, the following equation was used:


$$\frac{\left(\mathrm{Vessel}\;\mathrm{attenuation}-\mathrm{Background}\;\mathrm{attenuation}\right)}{\;\left(\mathrm{Background}\;\mathrm{noise}\right)}.$$

Circular regions of interest (ROIs) were used: a 5-mm ROI placed in the vessel lumen in the contrast media and a 30-mm ROI in the phantom background. Image noise was defined as the SD of the attenuation in the background ROI.

Further quantitative evaluation was performed using VMI from 40 to 190 keV in 10-keV increments. These VMI data sets were exported and analyzed in ImageJ. For each of these energy levels, the degree of luminal stenosis was assessed for all contrast media at the 3 diameter stenosis levels of 25%, 50%, and 75%. Stenosis measurements were obtained from attenuation profiles: for each calcified plaque, a single straight line was placed orthogonal to the vessel axis at the point of maximal narrowing (90 degrees to the centerline) to sample the full lumen cross-section and compute an attenuation profile. The exact line (identical coordinates/length) was reused across all reconstructions and energy levels to eliminate repositioning variability. Lumen diameters were read from the profile within the stenosis and at the proximal reference segment (using the constant window settings defined a priori), and the apparent percentage stenosis was calculated relative to proximal and distal reference diameters. Given the fixed phantom geometry and the deterministic, profile-based readout, each condition yields a single value; therefore, no within-condition SD applies. All measurements were performed by a cardiovascular radiology fellow.

Attenuation profiles were extracted along the vessel centerline of each vessel segment, allowing the contrast-enhanced lumen to be distinguished from the surrounding oil and calcifications based on their attenuation differences to allow the calculation of a percentage narrowing.

Constant window settings were applied for coronary stenosis measurements on CCTA images, regardless of the keV level, to be able to quantify exclusively the effect of the energy level on stenosis quantification. VMIs at 70 keV were chosen for defining the window settings. The window level corresponded to the mean attenuation measured by placing an ROI in the lumen of the 6 mm phantom. The window width was then obtained by multiplying the window level by 2.5 as previously recommended.^17–19^

### Qualitative Image Assessment

In addition to the quantitative measurements, a qualitative image analysis was performed across the entire energy range (40 to 190 keV). For each contrast medium, 2 independent, experienced cardiovascular radiologists, blinded to the quantitative results, rated image quality throughout the VMI energy levels using 3 criteria:

- subjective blooming artifacts, defined as apparent plaque enlargement or vessel lumen underestimation due to low attenuation contrast;
- perceived vessel-to-background contrast, assessed as the subjective conspicuity of the lumen relative to the surrounding matrix.
- subjective image noise, defined as the perceptible random variation in pixel values within a homogeneous region.

These criteria were scored on a 5-point visual analog scale (1=nondiagnostic, 5=excellent and image noise as 1=high noise, 5=minimal noise). The optimal keV setting for each contrast medium was defined as the energy level achieving the highest combined score across all 3 criteria. Discrepancies between readers were resolved by consensus.

### Statistical Analyses

All statistical analyses were performed using commercially available software (R, version 4.0; R Foundation for Statistical Computing). The Spearman rank correlation was used to assess the relationship between energy level (keV) and attenuation, CNR of the contrast medium, and stenosis quantification. Correlation coefficients (ρ) and *P* values were calculated, with a 2-tailed *P* value below 0.05 considered to indicate statistical significance.

## RESULTS

**FIGURE 2:**
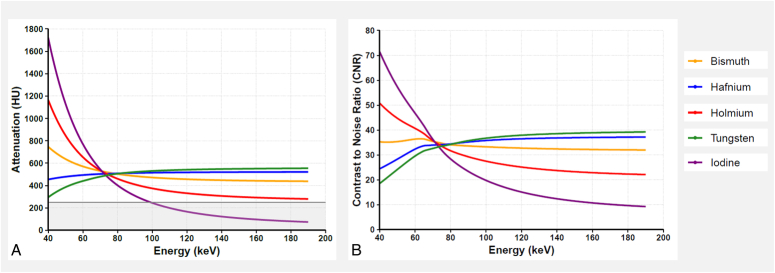
Attenuation (in Hounsfield units, HU) as a function of energy level (keV) for all 5 contrast media (A), and contrast-to-noise ratio between the surrounding tissue (oil) and all contrast media as a function of the virtual monoenergetic levels (keV) (B). The gray-shaded area below 250 HU demonstrates the reported minimal vascular attenuation for CT angiography.^16^

Quantitative evaluation demonstrated a significant dependency of contrast attenuation and CNR on both the VMI energy level and the type of contrast media (*P*<0.001) (Fig. 2).

### Vessel Attenuation

The attenuation behavior of all 5 contrast media varied markedly across the VMI energy levels. Iodine exhibited a steep decline in attenuation, from over 1700 HU at 40 keV to 250 HU at ~100 keV. Beyond 100 keV, the attenuation values of iodine fell beneath the predefined threshold of 250 HU.

Holmium and bismuth showed similar attenuation characteristics to iodine, though at a lower level at low VMI energy levels and higher attenuation levels at high VMI energy levels. In distinction, tungsten and hafnium increased attenuation with increasing VMI energy levels, demonstrating relative attenuation stability at higher keV. For instance, tungsten maintained ~554 HU at 190 keV, whereas iodine dropped to 74 HU at the same energy level (Table 1 and Fig. 2).

### Contrast-to-Noise Ratio

The CNR of iodine was highest at low keV (CNR 71 at 40 keV) but steadily decreased with increasing energy, reaching a CNR of 9 at 190 keV. In contrast, tungsten had a low CNR of 19 at 40 keV and increased continuously to 39 at 190 keV, reaching the highest CNR of all contrast media at VMI energy levels above 70 keV. Hafnium and bismuth showed similar patterns, while hafnium increased moderately from 24 CNR at 40 keV, bismuth stayed nearly consistent with 35 CNR at 40 keV to 32 at 190 keV, respectively. Holmium demonstrated a CNR decrease between the values of iodine and bismuth, starting at 51 at 40 keV and decreasing to 22 at 190 keV (Fig. 2).

### Stenosis Quantification

Across all diameter stenoses (25%, 50%, and 75%), quantification revealed a consistent trend of decreasing overestimation at increasing VMI energy levels. Overestimation was most pronounced from 40 to 60 keV. At 25% nominal stenosis, the measured stenosis was >50% at 40 keV, corresponding to a 25% overestimation, for all contrast media. Comparable overestimation was found for 50% and 75% nominal stenosis. At 190 keV, stenosis quantification was improved for all contrast media. Of all 5 media, tungsten, hafnium, and bismuth were the most accurate, reaching 0% to 2.5% overestimation. Iodine was the most inaccurate, with 10% to 13.5% (depending on the stenosis) above the real stenosis, due to an underestimation of the remaining lumen as the CNR considerably decreased.

**FIGURE 3:**
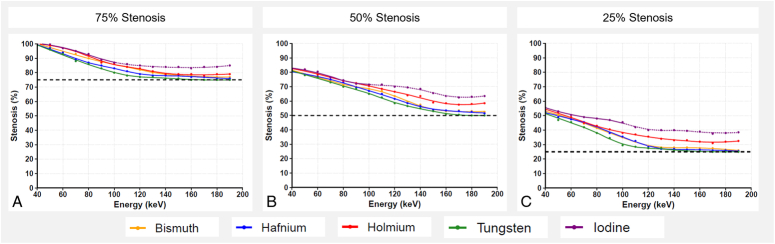
Stenosis quantifications on virtual monoenergetic images from 40 to 190 keV in 10-keV increments, shown for the vessels with 75% stenosis (A), 50% stenosis (B), and 25% stenosis (C). Averaged data from the 2 vessels (with 4 and 6 mm diameter, respectively) are shown because of the high agreement. Iodine is shown as a dashed line from 100 keV onward, as its attenuation falls below the minimum threshold required for CT angiography (Fig. 2).

This energy-dependent pattern of stenosis overestimation was consistently observed in both vessel sizes. We therefore averaged the results for clarity (Fig. 3).

### Image Quality

**FIGURE 4:**
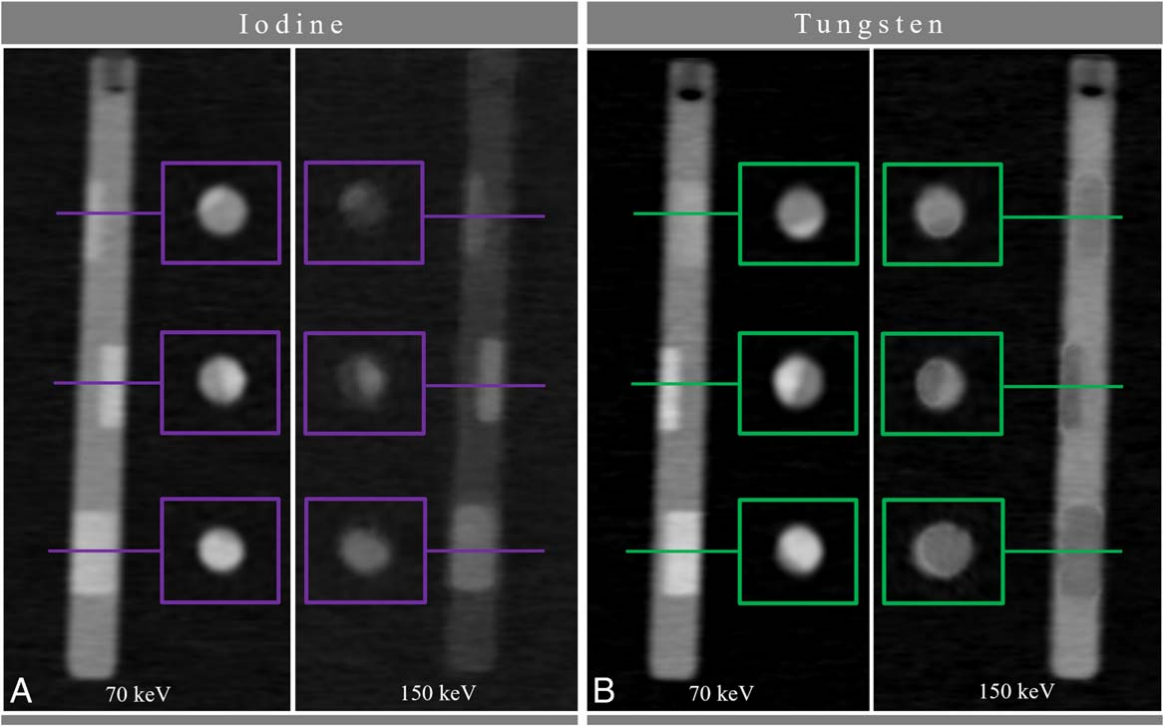
Vessel phantom with iodine (A) and tungsten (B), shown at 40 and 190 keV. Cross-sectional images are displayed for stenosis levels of 0%, 25%, 50%, and 75%. Window level and width were adapted manually [iodine: 525 (L)/ 1312 (W) and tungsten: 494 (L)/ 1235 (W)]. Note that longitudinal views are provided for orientation purposes only. In the phantom, each stenosis was rotated by 120 degrees, meaning that the long-axis slices do not depict all stenoses at their maximum diameter within this view.

Qualitative evaluation revealed that the optimal visual depiction of the coronary arteries depended on the contrast medium. In the iodine phantoms, blooming obscured the lumen boundaries at low VMI energy levels (Fig. 4 and Supplemental Figure, Supplemental Digital Content 1, http://links.lww.com/RLI/B83). Conversely, high VMI energy levels in combination with very high-Z contrast media allowed for a better separation between the calcified plaque and the lumen, even in the highest grade stenoses. Tungsten achieved the highest overall quality score (15/15) within the range of 140 to 160 keV, driven by minimal blooming, high vessel-to-background contrast, and low perceived noise. Hafnium, bismuth, and holmium showed peak scores of 14/15 (Ha), 12/15 (Bi), and 11/15 (Ho) at the same energy level as tungsten, offering a balanced reduction of blooming with excellent lumen conspicuity. Iodine achieved a lower peak score (9/15) between 55 and 70 keV, where contrast was adequate, but image noise was still relevant and blooming remained an issue. Across all contrast media, subjective image noise worsened progressively at higher keV settings, while blooming effects dominated at the lowest VMI energy levels.

## DISCUSSION

This experimental study with photon-counting detector CT demonstrated that high atomic number (*very high-Z*) contrast media offer potential advantages for quantifying stenosis caused by calcified plaques in small vessels when using VMI. High-energy VMIs reduce blooming artifacts from dense calcifications and thus enable more accurate stenosis quantification, while the vessel attenuation from very high-Z contrast media remains sufficient. In distinction, the attenuation of iodine drops below the 250 HU threshold, which is considered the minimum for adequate enhancement, precluding the usefulness of high-energy level VMI reconstructions in CT angiography with iodinated contrast media.

Accurate stenosis quantification from heavily calcified plaques remains a challenge due to calcium blooming artifacts, which are most pronounced on low-energy VMIs. These artifacts lead to significant overestimation of plaque volume and adjacent lumen narrowing.^9,20^ In our experimental phantom model, all contrast media substantially overestimated stenoses at 40 keV, up to 31% for the 25% stenosis using iodine. The use of VMI at higher energy levels considerably reduces calcium blooming artifacts.^10^ However, with iodine, vessel attenuation drops below 250 HU at ≥100 keV, thereby limiting its clinical utility. In addition, the low spectral property differences of calcium and iodine (k-edge at 33.2 keV) compared with very high-Z contrast media further reduced the differentiation.^12^ As a result, while calcium blooming artifacts diminish at higher VMI energy levels, the attenuation of iodine-based contrast media also decreases.

We have focused our experiments on VMI because this technique is clinically available today. Still, it must be acknowledged that K-edge imaging may offer greater potential, exploiting element-specific absorption edges (eg, Ho 55.6 keV, Hf 65.3 keV, W 69.5 keV, and Bi 90.5 keV) and the sharp increase in absorption for x-ray energies above the edge. Optimized adjustment of the energy thresholds of the PCD-CT (directly below and above the K-edge) can improve the CNR^21,22^ and enable material-specific imaging,^23^ although the spectral separation is not perfect due to the spectral overlap of the energy bins. By using more than 2 energy thresholds, different K-edge contrast agents can be separated in material-specific images, opening further applications. The implementation of PCDs with multiple adjustable thresholds for routine clinical use could accelerate the development and clinical approval of high-Z contrast media,^12^ which are currently not clinically available. Our VMI-based results represent a pragmatic short-term approach to demonstrate their advantages, with K-edge imaging as a complementary, longer-term avenue.

Very high-Z contrast media exhibit more distinct spectral properties than calcium. At elevated VMI energy levels, agents such as tungsten (k-edge at 69.5 keV) and hafnium (k-edge at 65.3 keV) maintained high attenuation and reduced stenosis overestimation to below 2.5% at 190 keV. Notably, tungsten preserved attenuation values of ~554 HU at 190 keV, compared with only 74 HU for iodine, underscoring the ability of very high-Z media to sustain vessel conspicuity at VMI energy levels. This stability in attenuation at elevated VMI energy levels contributed to improved lumen definition and more accurate stenosis quantification in the presence of calcified plaques. Holmium showed intermediate performance in both attenuation and CNR, likely reflecting its K-edge position at 55.6 keV and corresponding spectral behavior, which places it between iodine and other very high-Z media such as tungsten or bismuth.

The observed effects of reduced blooming at higher VMI energy levels are not only true for calcified plaques but may also be observed for coronary stents.^11^ Blooming artifacts from stent struts lead to a reduced in-stent lumen visualization, often precluding the ability to rule-in or rule-out in-stent restenosis.^24^ Thus, the herein investigated high Z-atomic number contrast media could also contribute to improved coronary stent imaging by maintaining high vessel lumen attenuation and CNR at elevated VMI energy levels.

Another potential advantage of very high-Z contrast media is their ability to offer prolonged circulation times, particularly when formulated as liposomal or nanoparticle-bound media.^25,26^ Unlike iodine, which is rapidly cleared via renal excretion, these media can persist longer in the vascular system, potentially providing a broader imaging window and enabling multiphase or delayed imaging protocols. Such properties could, in principle, be advantageous for applications such as blood-pool imaging, myocardial perfusion CT, or endoleak detection, where a stable vascular signal is desirable.^21^ If combined with suitable delivery strategies, the extended residence time might also allow for lower total contrast volumes, as a sustained intravascular signal could reduce the need for high-rate bolus injections.^27^ This can become especially valuable for patients with impaired renal function, for whom contrast minimization is critical. Moreover, the ability to decouple scan timing from peak arterial enhancement could simplify workflow in clinical practice.

The development of contrast agents with higher atomic numbers could bring significant advantages for CT imaging. However, there are numerous hurdles to overcome:^12^ a concentration similar to iodine-containing contrast agents must be reached to ensure sufficient contrast in the CT image. Solubility must be high to enable low viscosity and osmolality in the final formulation. The molecules must be very stable and, to ensure good tolerability, must not be metabolized by the body to ensure rapid and complete renal excretion. A new generation of tungsten compounds (ie, multi-dental tungsten clusters) demonstrated many of these properties:^28^ They were well tolerated and highly soluble, with tungsten concentrations similar to the iodine concentrations of commercial contrast agents. However, for various reasons, no contrast agent with a high atomic number has yet reached the market. The highly optimized properties of iodine-containing contrast agents, and particularly their cost-effective production, have proven to be significant hurdles for the transition of novel contrast agents to clinical application.^22^

We must acknowledge several study limitations. First, we used a static coronary phantom without cardiac motion, modeling only uniform calcified stenoses and vessels with a uniform diameter, which does not reflect the complexity of in vivo coronary anatomy. Patient-related factors such as cardiac motion, vessel tortuosity, and heterogeneous plaque morphology were not considered. Second, only one CT system and a single reconstruction algorithm were used, potentially limiting generalizability. Alternative imaging approaches, such as the ultra-high-resolution mode^17^ or spectral virtual non-calcium reconstructions,^23^ as well as larger reconstruction matrices (eg, 1024 ×1024) were not evaluated. Therefore, potential effects on in-plane resolution, noise, and stenosis quantification remain unknown, noting that a larger matrix at the same reconstruction diameter reduces pixel size (higher nominal in-plane resolution) but typically increases per-pixel noise and can slightly alter CNR/edge profiles with modest impact on attenuation-profile–based diameter measurements. Third, the phantom incorporated a single plaque density (calcium hydroxyapatite ≈794 HU at 120 kVp) to standardize conditions. Consequently, our results may not generalize to plaques with different mineral densities or compositions, for which attenuation profiles, blooming magnitude, and stenosis overestimation could differ. Future work should validate these findings across multiple CaHA concentrations and mixed plaque surrogates to better reflect in vivo variability. Fourth, only one identical concentration of each contrast medium was tested, which does not account for possible performance differences at other clinically relevant concentrations. Moreover, the VMI settings used in this study were optimized for iodine and not for the novel very high-Z contrast media investigated. This likely leads to an underestimation of their potential performance and should be addressed in future work. Fifth, fixed window settings were applied for all analyses. However, this was intentionally done to isolate and to quantify the exclusive effect of energy level on coronary stenosis assessment without confounding from windowing adjustments. Finally, while very high-Z media show theoretical promise, they remain far from clinical implementation, and our findings should therefore be interpreted within an experimental and preclinical context.^14,15^

In conclusion, our experimental study supports the notion that very high-Z contrast media provide superior lumen definition and more accurate stenosis assessment using VMI at high energy levels compared with iodine, since high keV monoenergetic images minimize calcium blooming. Further research is warranted to translate these potential advantages into clinical practice.

## Supplementary Material
