# Supplementary material for: High-Z Contrast Media for Coronary Photon-counting Detector CT Angiography: Improved Quantification of Calcified Stenoses
Source: Invest Radiol. 2025 Dec 1;61(8):568–74. doi: 10.1097/RLI.0000000000001262 (PMC13317894; doi:10.1097/RLI.0000000000001262)

Supplemental Figure: Overview of the calcified 50% diameter stenosis with all five contrast agents for energy levels between 40 and 190 keV with fixed window levels. Bismuth: 520 (L)/ 1300 (W), Hafnium: 515 (L)/ 1287 (W), Holmium: 537 (L)/ 1342 (W) Iodine: 525 (L)/ 1312 (W) and Tungsten: 494 (L)/ 1235 (W).

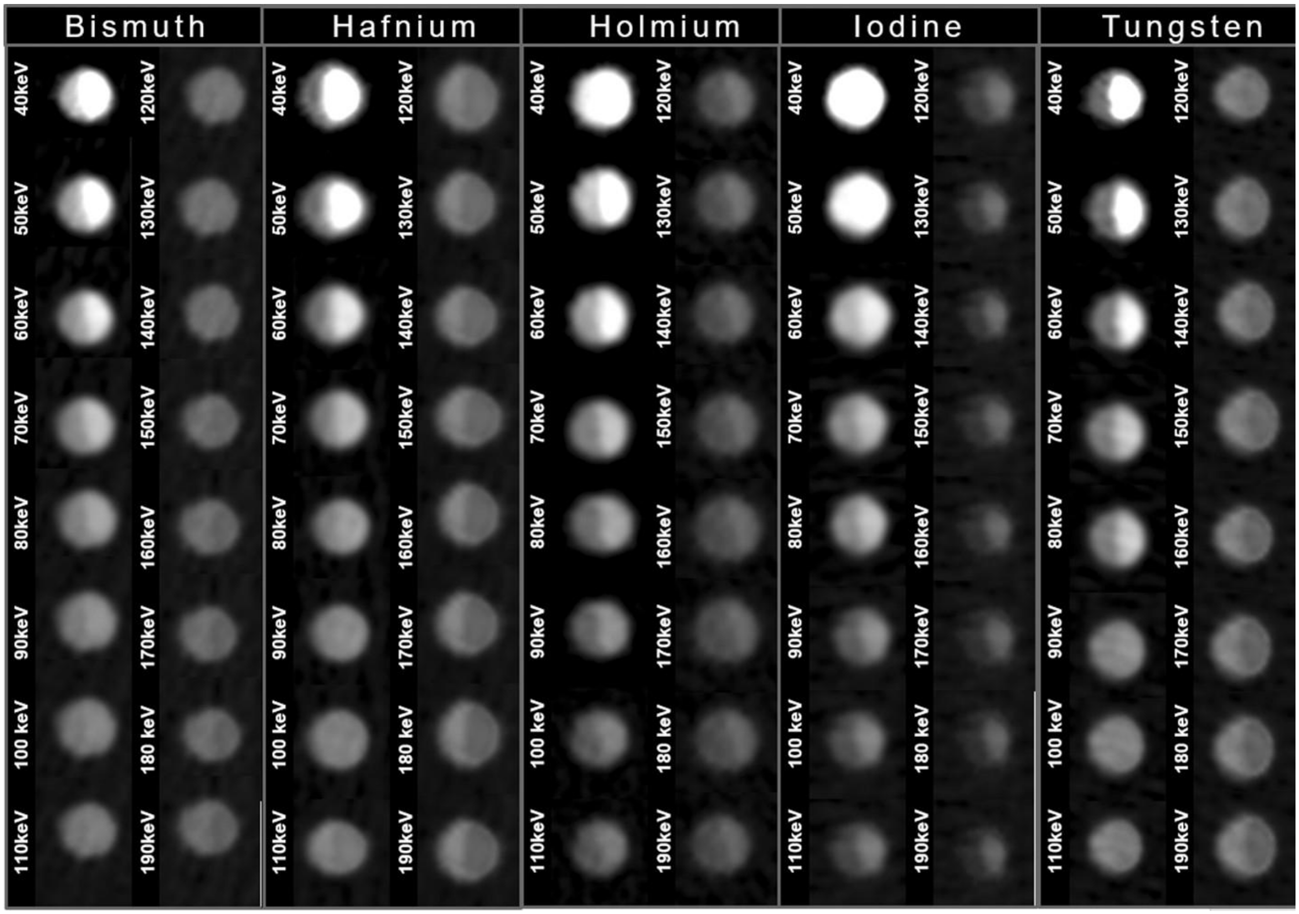

Supplement: Supplementary file 1 [file rli-61-568-s001.pdf]
